# Supplementary material for: HIF-1 Interacts with TRIM28 and DNA-PK to release paused RNA polymerase II and activate target gene transcription in response to hypoxia
Source: Nat Commun. 2022 Jan 14;13:316. doi: 10.1038/s41467-021-27944-8 (PMC8760265; doi:10.1038/s41467-021-27944-8)
Supplement: Supplementary file 3 — Description of additional Supplementary File [file 41467_2021_27944_MOESM3_ESM.pdf]

Description of additional Supplementary data files

Supplementary Data 1: Identification of HIF-dependent hypoxia-induced and repressed genes by RNA sequencing. RNA-seq data from SUM159 breast cancer cells revealed that exposure to hypoxia (24 hours at 1% O_2) significantly increased the expression of 1,307 RNAs and decreased the expression of 817 RNAs in a HIF-dependent manner.
