# Supplementary material for: HIF-1 Interacts with TRIM28 and DNA-PK to release paused RNA polymerase II and activate target gene transcription in response to hypoxia
Source: Nat Commun. 2022 Jan 14;13:316. doi: 10.1038/s41467-021-27944-8 (PMC8760265; doi:10.1038/s41467-021-27944-8)
Supplement: Supplementary file 5 — Reporting summary [file 41467_2021_27944_MOESM5_ESM.pdf]

nature portfolio

Corresponding author(s): Gregg L. Semenza

Last updated by author(s): Nov 22, 2021

Reporting Summary

Nature Portfolio wishes to improve the reproducibility of the work that we publish. This form provides structure for consistency and transparency in reporting. For further information on Nature Portfolio policies, see our Editorial Policies and the Editorial Policy Checklist.

Statistics

For all statistical analyses, confirm that the following items are present in the figure legend, table legend, main text, or Methods section.

n/a	Confirmed	
☐	☒	The exact sample size (*n*) for each experimental group/condition, given as a discrete number and unit of measurement
☐	☒	A statement on whether measurements were taken from distinct samples or whether the same sample was measured repeatedly
☐	☒	The statistical test(s) used AND whether they are one- or two-sided<br>*Only common tests should be described solely by name; describe more complex techniques in the Methods section.*
☐	☒	A description of all covariates tested
☐	☒	A description of any assumptions or corrections, such as tests of normality and adjustment for multiple comparisons
☐	☒	A full description of the statistical parameters including central tendency (e.g. means) or other basic estimates (e.g. regression coefficient) AND variation (e.g. standard deviation) or associated estimates of uncertainty (e.g. confidence intervals)
☐	☒	For null hypothesis testing, the test statistic (e.g. *F*, *t*, *r*) with confidence intervals, effect sizes, degrees of freedom and *P* value noted<br>*Give P values as exact values whenever suitable.*
☒	☐	For Bayesian analysis, information on the choice of priors and Markov chain Monte Carlo settings
☒	☐	For hierarchical and complex designs, identification of the appropriate level for tests and full reporting of outcomes
☒	☐	Estimates of effect sizes (e.g. Cohen's *d*, Pearson's *r*), indicating how they were calculated

Our web collection on statistics for biologists contains articles on many of the points above.

Software and code

Policy information about availability of computer code

Data collection	Bio-Rad CFX manager (Version 3.1) for quantitative PCR; Bio-Rad Image Lab (Version 4.1) for Western Blot; Agilent Seahorse Wave Software (Version 2.6) for Seahorse assay.

Data analysis

RIME:
Tandem mass spectra were extracted by Proteome Discoverer version 2.3 (Thermo Fisher Scientific) and searched against the SwissProt_Full_Synchronized_2018_08 database using Mascot version 2.6.2 (Matrix Science). Mascot ".dat" files were compiled in Scaffold version 3 (Proteome Software) to validate MS/MS-based peptide and protein identifications. Peptide identifications were accepted if false discovery rate (FDR) was less than 1%, based on a concatenated decoy database search by the Peptide Prophet algorithm with Scaffold delta-mass correction.

RNA-Sequencing:
RNA-seq data were processed and interpreted using Genialis Expressions software version (https://www.genialis.com). The automated data analysis on the Genialis platform consisted of the following steps: sequence quality checks were performed on raw and trimmed reads (FastQC, Version 0.11.9), trimming and quality filtering of reads (BBDuk, Version 38.46), mapping to reference human genome Ensembl v.92 (STAR, Version 2.5.3a), expression quantification (featureCounts, Version 2.0.2), and expression normalisation (RNA.norm, Version 1.12.0). Key QC metrics (e.g. mapping statistics) were collected. As an additional quality control step, a sample of one million reads (Seqtk tool, Version 1.3) was mapped (STAR, Version 2.5.3a) separately to human rRNA and globin sequences to evaluate the proportion of these reads in the sample. Differential gene expression analyses were performed with DESeq2. Lowly-expressed genes, which have expression count summed over all samples below 10, were filtered out from the differential expression analysis input matrix (https://www.genialis.com/). Differential expression results with FDR < 0.05 and mRNA fold change > 1.5 were used as cutoff for further analysis.

GraphPad Prism 8 (GraphPad Inc) was used for graph generation and data analyses.

For manuscripts utilizing custom algorithms or software that are central to the research but not yet described in published literature, software must be made available to editors and reviewers. We strongly encourage code deposition in a community repository (e.g. GitHub). See the Nature Portfolio guidelines for submitting code & software for further information.

Data

Policy information about availability of data

All manuscripts must include a data availability statement. This statement should provide the following information, where applicable:

- Accession codes, unique identifiers, or web links for publicly available datasets
- A description of any restrictions on data availability
- For clinical datasets or third party data, please ensure that the statement adheres to our policy

The RNA sequencing data generated in this study have been deposited in the Gene Expression Omnibus (GEO) under accession codes GSE167956 [https://www.ncbi.nlm.nih.gov/geo/query/acc.cgi?acc=GSE167956]. The mass spectrometry proteomics data have been deposited to the ProteomeXchange Consortium with the data set identifier PXD024373 [http://www.ebi.ac.uk/pride/archive/projects/PXD024373]. Public TCGA data generated by the TCGA Research Network are available at [https://www.cancer.gov/tcga]. All other data supporting the findings of this study are available in the Article, Supplementary Information or from the corresponding author upon reasonable request. Source data are provided with this paper.

Field-specific reporting

Please select the one below that is the best fit for your research. If you are not sure, read the appropriate sections before making your selection.

[x] Life sciences [ ] Behavioural & social sciences [ ] Ecological, evolutionary & environmental sciences

For a reference copy of the document with all sections, see nature.com/documents/nr-reporting-summary-flat.pdf

Life sciences study design

All studies must disclose on these points even when the disclosure is negative.

Sample size	Sample sizes were chosen according to the standards of the field. For in vitro experiments, at least three independent biological replicates was performed to allow for proper statistical analyses of the data. No in vivo experiments were performed in this study.
Data exclusions	No data exclusion was performed.
Replication	All experiments were repeated at least 3 times in different biological replicates, unless stated differently in figure legends.
Randomization	No animal subjects were used in the study. For in vitro experiments, cells were randomly allocated into control and experimental groups.
Blinding	No animal subjects were used in the study. In vitro experiments, cell culture were not blinded, since the respective genetic manipulations had to be induced by the investigator and therefore could not be blinded. For other experiments, such as biochemical experiments, investigators were not blinded to the identity of samples to ensure appropriate data collection and and analysis. And all the control and experimental samples were processed in parallel to ensure consistent conditions.

Reporting for specific materials, systems and methods

We require information from authors about some types of materials, experimental systems and methods used in many studies. Here, indicate whether each material, system or method listed is relevant to your study. If you are not sure if a list item applies to your research, read the appropriate section before selecting a response.

Materials & experimental systems

n/a	Involved in the study
	x Antibodies
	x Eukaryotic cell lines
x	Palaeontology and archaeology
x	Animals and other organisms
x	Human research participants
x	Clinical data
x	Dual use research of concern

Methods

n/a	Involved in the study
x	ChIP-seq
x	Flow cytometry
x	MRI-based neuroimaging

Antibodies

Antibodies used

HIF-1α, Novus Biologicals (NB100-479), Chromatin Immunoprecipitation;
HIF-1α, Cayman Chemical (10006421), Immunoprecipitation;
HIF-1α, BD Biosciences (610959), Western blotting;
HIF-2α, Novus Biologicals (NB100-122), Chromatin Immunoprecipitation/Western blotting;
HIF-1β, Novus Biologicals (NB100-110), Chromatin Immunoprecipitation/Western blotting;
TRIM28-pSer824, Novus Biologicals (NB100-2350), Chromatin Immunoprecipitation/Western blotting/Immunoprecipitation;
TRIM28, Novus Biologicals (NB500-158), Chromatin Immunoprecipitation/Immunoprecipitation/Western blotting;
DNA-PKcs, Novus Biologicals (NB100-658), Chromatin Immunoprecipitation/Western blotting;
DNA-PKcs-pSer2056, Abcam (ab18192), Western blotting;
DNA-PKcs-pThr2609, Novus Biologicals (NBP1-02456), Western blotting/Chromatin Immunoprecipitation;
KU70, Novus Biologicals (NB100-1915), Chromatin Immunoprecipitation/Immunoprecipitation/Western blotting;
KU86, Novus Biologicals (NB100-508), Chromatin Immunoprecipitation/Immunoprecipitation/Western blotting;
ATM, Novus Novus Biologicals (NB100-309), Western blotting;
ATM-pS1981, Novus Biologicals (NB100-306), Western blotting;
Histone H2AX, Novus Biologicals (NB100-383), Western blotting;
Histone γH2AX, Novus Biologicals (NB100-384), Western blotting/Chromatin Immunoprecipitation;
CDK9, Novus Biologicals (NBP2-67811), Chromatin Immunoprecipitation/Western blotting;
CDK9, Novus Biologicals (H00001025-M07), Immunoprecipitation/Western blotting;
NELF-E, Santa Cruz Biotechnology (sc-377052), Chromatin Immunoprecipitation;
H3K4me3, Novus Biologicals (NB21-1023), Chromatin Immunoprecipitation;
H3K36me3, Novus Biologicals (NBP2-59199), Chromatin Immunoprecipitation;
Histone H3, Novus Biologicals (NB500-267), Chromatin Immunoprecipitation;
RNA Polymerase II-pSer2, Novus Biologicals (NBP2-59215), Chromatin Immunoprecipitation;
RNA Polymerase II-pSer5, Novus Biologicals (NBP2-59219), Chromatin Immunoprecipitation;
RNA Polymerase II, Novus Biologicals (NBP2-32080), Chromatin Immunoprecipitation;
FLAG epitope tag, MilliporeSigma (F7425), Immunoprecipitation;
Actin, Santa Cruz Biotechnology (sc-47778), Western blotting;
FLAG epitope tag, MilliporeSigma (F3165), Western blotting;
HA epitope tag, Novus Biologicals (NB600-363), Western blotting;
HA epitope tag, Santa Cruz Biotechnology (sc-7392), Immunoprecipitation;
myc epitope tag, Novus Biologicals (NB600-302), Western blotting.

Validation

All the antibodies used in this study were bought commercially and validated by the manufacturer and/or other published studies. The detailed information can be found on the manufacturers' websites through the catalog numbers listed above. we also provided the validation of the antibodies for the species and application in our experiments. For example, we validated HIF-1α (BD Biosciences, 610959), HIF-2α (Novus Biologicals, NB100-122), TRIM28 (Novus Biologicals, NB500-158), KU70 (Novus Biologicals, NB100-1915), KU86 (Novus Biologicals, NB100-508) and DNA-PKcs (Novus Biologicals, NB100-658) by RNAi technology in MDA-MB-231 and SUM159 human breast cancer cell lines. For other antibodies:
HIF-1α, Novus Biologicals (NB100-479): website validation, 361 citations, successfully used for ChIP in various cell lines;
HIF-1α, Cayman Chemical (10006421): website validation, 106 citations, Western blot/IP analysis of various cell lines successfully stained;
HIF-1α, BD Biosciences (610959): website validation, 254 citations, Western blot analysis of various cell lines successfully stained;
HIF-2α, Novus Biologicals (NB100-122): website validation, 655 citations, Western blot/ChIP analysis of various cell lines successfully stained;
HIF-1β, Novus Biologicals (NB100-110): website validation, 51 citations, Western blot/ChIP analysis of various cell lines successfully stained;
TRIM28-pSer824, Novus Biologicals (NB100-2350): website validation, 30 citations, Western blot/IP analysis of various cell lines successfully stained;
TRIM28, Novus Biologicals (NB500-158): website validation, 15 citations, Western blot/IP analysis of various cell lines successfully stained;
DNA-PKcs, Novus Biologicals (NB100-658): website validation, 1 citations, successfully used in Western blot assay (PMID: 28132842);
DNA-PKcs-pSer2056, Abcam (ab18192): website validation, 168 citations, Western blot analysis of various cell lines successfully stained;
DNA-PKcs-pThr2609, Novus Biologicals (NBP1-02456): website validation, 2 citations, successfully used in Western blot assay (PMID:

23691119);
KU70, Novus Biologicals (NB100-1915): website validation, 3 citations, successfully used in Western blot assay, also validated in this study by RNAi in MDA-MB-231 and SUM159 human breast cancer cell lines;
KU86, Novus Biologicals (NB100-508): website validation, 3 citations, successfully used in Western blot assay, also validated in this study by RNAi in MDA-MB-231 and SUM159 human breast cancer cell lines;
ATM, Novus Biologicals (NB100-309): website validation, 121 citations, Western blot analysis of various cell lines successfully stained;
ATM-pS1981, Novus Biologicals (NB100-306): website validation, 24citations, Western blot analysis of various cell lines successfully stained;
Histone H2AX, Novus Biologicals (NB100-383): website validation, 11 citations, successfully used for Western blot in various cell lines;
Histone γH2AX, Novus Biologicals (NB100-384): website validation, 108 citations, successfully used for Western blot/ChIP assay in various cell lines.
CDK9, Novus Biologicals (NBP2-67811): website validation, 108 citations, Western blot/IP/ChIP analysis of various cell lines successfully stained;
CDK9, Novus Biologicals (H00001025-M07): website validation, 1 citations, successfully used for Western blot/IP (PMID: 28754981);
NELF-E, Santa Cruz Biotechnology (sc-377052): website validation, 7 citations, successfully used for Western blot and ChIP assay (PMID: 32402252);
H3K4me3, Novus Biologicals (NB21-1023): website validation, 4 citations, successfully used in ChIP-qPCR assay (PMID: 34452909)
H3K36me3, Novus Biologicals (NBP2-59199): successfully used in ChIP-qPCR assay (PMID: 34452909);
Histone H3, Novus Biologicals (NB500-267): website validation, 3 citations, successfully used for Western blot or ChIP assay (PMID: 29629903);
RNA Polymerase II-pSer2, Novus Biologicals (NBP2-59215): successfully used in ChIP-qPCR assay (PMID: 34452909);
RNA Polymerase II-pSer5, Novus Biologicals (NBP2-59219): successfully used in ChIP-qPCR assay (PMID: 34452909);
RNA Polymerase II, Novus Biologicals (NBP2-32080): website validation, 2 citations, successfully used in ChIP assay (PMID: 31347271);
FLAG epitope tag, MilliporeSigma (F7425): website validation, 2291 citations, Western blot and IP analysis of various cell lines successfully stained;
Actin, Santa Cruz Biotechnology (sc-47778): website validation, 8871 citations, Western blot analysis of various cell lines successfully stained;
FLAG epitope tag, MilliporeSigma (F3165): website validation, 7594 citations, Western blot analysis of various cell lines successfully stained;
HA epitope tag, Novus Biologicals (NB600-363): website validation, 77 citations, Western blot analysis of various cell lines successfully stained;
HA epitope tag, Santa Cruz Biotechnology (sc-7392): website validation, 1831 citations, Western blot and IP analysis of various cell lines successfully stained;
myc epitope tag, Novus Biologicals (NB600-302): website validation, 31 citations, Western blot analysis of various cell lines successfully stained.

Eukaryotic cell lines

Policy information about cell lines

Cell line source(s)	MDA-MB-231 and SUM159 cells were obtained from ATCC.
Authentication	Breast cancer cell lines were authenticated by short tandem repeat DNA profiling analysis.
Mycoplasma contamination	All cell lines are tested mycoplasma regularly and the cell lines used in the study are mycoplasma free.
Commonly misidentified lines (See ICLAC register)	No commonly misidentified cell lines were used.
