# Supplementary material for: HIF-1 Interacts with TRIM28 and DNA-PK to release paused RNA polymerase II and activate target gene transcription in response to hypoxia
Source: Nat Commun. 2022 Jan 14;13:316. doi: 10.1038/s41467-021-27944-8 (PMC8760265; doi:10.1038/s41467-021-27944-8)
Supplement: Supplementary file 1 — Supplementary Information [file 41467_2021_27944_MOESM1_ESM.docx]

**Supplementary Information**

**HIF-1 Interacts with TRIM28 and DNA-PK to Release Paused RNA Polymerase II and Activate Target Gene Transcription in Response to Hypoxia**

Yongkang Yang^1,2^, Haiquan Lu^1,2^, Chelsey Chen^1,3^, Yajing Lyu^1^, Robert N. Cole^4^ and Gregg L. Semenza^1,2,4,5^

^1^Institute for Cell Engineering, Johns Hopkins University School of Medicine, Baltimore, MD, USA. ^2^Sidney Kimmel Comprehensive Cancer Center at Johns Hopkins, Baltimore, MD, USA. ^3^Johns Hopkins University, Baltimore, MD, USA. ^4^Department of Biological Chemistry, Johns Hopkins University School of Medicine, Baltimore, MD, USA. ^5^McKusick-Nathans Department of Genetic Medicine, Johns Hopkins University School of Medicine, Baltimore, MD 21205, USA.

Correspondence: gsemenza@jhmi.edu (G.L.S)

**Supplementary Table 1-6**

**Supplementary Figure 1-8**

**Supplementary Table 1. Antibodies used for ChIP.**

| **Antibody** | **Source** | **Catalog #** | **Usage** |
| --- | --- | --- | --- |
| HIF-1α | Novus Biologicals | NB100-479 | 2 μg |
| HIF-2α | Novus Biologicals | NB100-122 | 2 μg |
| HIF-1β | Novus Biologicals | NB100-110 | 2 μg |
| TRIM28-pSer824 | Novus Biologicals | NB100-2350 | 2 μg |
| TRIM28 | Novus Biologicals | NB500-158 | 2 μg |
| DNA-PKcs | Novus Biologicals | NB100-658 | 2 μg |
| KU70 | Novus Biologicals | NB100-1915 | 2 μg |
| KU86 | Novus Biologicals | NB100-508 | 2 μg |
| CDK9 | Novus Biologicals | NB2-67811 | 2 μg |
| NELF-E | Santa Cruz Biotechnology | sc-377052 | 2 μg |
| H3K4me3 | Novus Biologicals | NB21-1023 | 2 μg |
| H3K36me3 | Novus Biologicals | NBP2-59199 | 2 μg |
| Histone H3 | Novus Biologicals | NB500-267 | 2 μg |
| RNA Polymerase II-pS2 | Novus Biologicals | NBP2-59215 | 2 μg |
| RNA Polymerase II-pS5 | Novus Biologicals | NBP2-59219 | 2 μg |
| RNA Polymerase II | Novus Biologicals | NBP2-32080 | 2 μg |
| DNA-PKcs-pT2609 | Novus Biologicals | NBP1-02456 | 2 μg |
| Histone γH2AX | Novus Biologicals | NB100-384 | 2 μg |

**Supplementary Table 2. Nucleotide sequence of ChIP-qPCR primers.**

| **Gene** | **Nucleotide sequence (5' to 3')** |
| --- | --- |
| ANGPTL4 | Forward: ATTTGCTGTCCTGGCATC |
|  | Reverse: CCAGCTCATTCTCTGGAATC |
| PDK1 | Forward: CGCGTTTGGATTCCGTG |
|  | Reverse: CCAGTTATAATCTGCCTTCCCTATTATC |
| RPL13A | Forward: GAGGCGAGGGTGATAGAG |
|  | Reverse: ACACACAAGGGTCCAATTC |
| CA9 | Forward: GACAAACCTGTGAGACTTTGGCTCC |
|  | Reverse: AGTGACAGCAGCAGTTGCACAGTG |
| PGK1 | Forward: TCTCGCACATTCTTCACGTCCGTT |
|  | Reverse: TAGTGAGACGTGCGGCTTCCGTTT |
| PGF | Forward: GCGGGTCTCGAACTCCTAAT |
|  | Reverse: TGGTAGCAATTGATCACGATT |
| SLC2A3 | Forward: TCAAGTCTTCAAGAAAGATCTAGG |
|  | Reverse: GACCCAGAGATGCTGTAATG |
| LDHA | Forward: TTGGAGGGCAGCACCTTACTTAGA |
|  | Reverse: GCCTTAAGTGGAACAGCTATGCTGAC |
| PKM2 | Forward: TTCCTGCCTCTTGGTATGAC |
|  | Reverse: CGGCTTGTTCCCTCCTAC |
| VEGFA | Forward: CAGACTCCACAGTGCATAC |
|  | Reverse: AGTTTGTGGAGCTGAGAAC |
| LOX | Forward: GAAGATTTCTCCTTCCCTCAC |
|  | Reverse: GAAGCGCATCACTCCTTT |

**Supplementary Table 3. Antibodies used for IP.**

| **Antibody** | **Source** | **Catalog #** | **Usage** |
| --- | --- | --- | --- |
| HIF-1α | Cayman Chemical | 10006421 | 2 μg |
| TRIM28 | Novus Biologicals | NB500-158 | 2 μg |
| KU70 | Novus Biologicals | NB100-1915 | 2 μg |
| KU86 | Novus Biologicals | NB100-508 | 2 μg |
| FLAG epitope tag | MilliporeSigma | F7425 | 2 μg |
| CDK9 | Novus Biologicals | H00001025-M07 | 2 μg |
| HA epitope tag | Santa Cruz Biotechnology | sc-7392 | 2 μg |

**Supplementary Table 4. Antibodies used for immunoblot assays.**

| **Primary Antibody** | **Source** | **Catalog #** | **Usage** |
| --- | --- | --- | --- |
| HIF-1α | BD Biosciences | 610959 | 1:500 |
| HIF-2α | Novus Biologicals | NB100-122 | 1:1000 |
| HIF-1β | Novus Biologicals | NB100-110 | 1:1000 |
| Actin | Santa Cruz Biotechnology | sc-47778 | 1:5000 |
| FLAG epitope tag | MilliporeSigma | F3165 | 1:3000 |
| HA epitope tag | Novus Biologicals | NB600-363 | 1:2000 |
| myc epitope tag | Santa Cruz Biotechnology | sc-40 | 1:1000 |
| TRIM28-pS824 | Novus Biologicals | NB100-2350 | 1:500 |
| TRIM28 | Novus Biologicals | NB500-158 | 1:3000 |
| DNA-PKcs | Novus Biologicals | NB100-658 | 1:1000 |
| DNA-PKcs-pS2056 | Abcam | ab18192 | 1:500 |
| DNA-PKcs-pT2609 | Novus Biologicals | NBP1-02456 | 1:500 |
| KU70 | Novus Biologicals | NB100-1915 | 1:3000 |
| KU86 | Novus Biologicals | NB100-508 | 1:3000 |
| CDK9 | Novus Biologicals | NB2-67811 | 1:1000 |
| CDK9 | Novus Biologicals | H00001025-M07 | 1:1000 |
| ATM | Novus Biologicals | NB100-309 | 1:1000 |
| ATM-pS1981 | Novus Biologicals | NB100-306 | 1:500 |
| Histone H2AX | Novus Biologicals | NB100-383 | 1:1000 |
| Histone γH2AX | Novus Biologicals | NB100-384 | 1:500 |
| **Secondary Antibody** | **Source** | **Catalog #** | **Usage** |
| Mouse IgG HRP | GE Healthcare | NA931V | 1:2000 |
| Rabbit IgG HRP | GE Healthcare | NA934V | 1:2000 |

**Supplementary Table 5. shRNA nucleotide sequences encoded in lentiviral vectors.**

| TRIM28  #1 | CCGGCCTGGCTCTGTTCTCTGTCCTCTCGAGAGGACAGAGAACAGAGCCAGGTTTTT |
| --- | --- |
| TRIM28  #2 | CCGGCTGAGACCAAACCTGTGCTTACTCGAGTAAGCACAGGTTTGGTCTCAGTTTTT |
| TRIM28  #3 | CCGGGACCACCAGTACCAGTTCTTACTCGAGTAAGAACTGGTACTGGTGGTCTTTTT |
| TRIM28  #4 | CCGGGCACTAGCTGTGAGGATAATGCTCGAGCATTATCCTCACAGCTAGTGCTTTTTG |
| TRIM28  #5 | CCGGATGGTGAACGTACTGTCTATTCTCGAGAATAGACAGTACGTTCACCATTTTTTG |
| DNA-PKcs #1 | CCGGGAAACAGCTGTCTCCGTAAATCTCGAGATTTACGGAGACAGCTGTTTCTTTTTTG |
| DNA-PKcs #2 | CCGGGCATTGAATTTGGGATAACTTCTCGAGAAGTTATCCCAAATTCAATGCTTTTTTG |
| DNA-PKcs #3 | CCGGCCTCCAGGTTAGGATTAATTGCTCGAGCAATTAATCCTAACCTGGAGGTTTTTTG |
| DNA-PKcs #4 | CCGGCCATCCCTTATAGGTTAATATCTCGAGATATTAACCTATAAGGGATGGTTTTTTG |
| DNA-PKcs #5 | CCGGCCTGAAGTCTTTACAACATATCTCGAGATATGTTGTAAAGACTTCAGGTTTTTTG |
| KU70  #1 | CCGGAAGAGTCTACCCGACATAAGCTCGAGCTTATGTCGGGTAGACTCTTCTTTTTG |
| KU70  #2 | CCGGGATGAGTCATAAGAGGATCATCTCGAGATGATCCTCTTATGACTCATCTTTTTG |
| KU70  #3 | CCGGCGTCAGATTATACTGGAGAAACTCGAGTTTCTCCAGTATAATCTGACGTTTTTG |
| KU70  #4 | CCGGCGTCAGATTATACTGGAGAAACTCGAGTTTCTCCAGTATAATCTGACGTTTTTG |
| KU70  #5 | CCGGCGACATAAGTCGAGGGACTTTCTCGAGAAAGTCCCTCGACTTATGTCGTTTTTG |
| KU86  #1 | CCGGAATCTAAGAGAGCTGCCATCGCTCGAGCGATGGCAGCTCTCTTAGATTTTTTTG |
| KU86  #2 | CCGGAGAGGAAGCCTCTGGAAGTTCCTCGAGGAACTTCCAGAGGCTTCCTCTTTTTTG |
| KU86  #3 | CCGGCGTGGGCTTTACCATGAGTAACTCGAGTTACTCATGGTAAAGCCCACGTTTTTG |
| KU86  #4 | CCGGCCTCATATCAAGCATAACTATCTCGAGATAGTTATGCTTGATATGAGGTTTTTG |
| KU86  #5 | CCGGAGAGGAAGCCTCTGGAAGTTCCTCGAGGAACTTCCAGAGGCTTCCTCTTTTTTG |

**Supplementary Table 6. Nucleotide sequences of primers used for RT-qPCR.**

| **RNA** | **Nucleotide sequence (5' to 3')** |
| --- | --- |
| ANGPTL4 mRNA  middle | Forward: GTCCACCGACCTCCCGTTA |
|  | Reverse: CCTCATGGTCTAGGTGCTTGT |
| ANGPTL4 mRNA  5’ end | Forward: AAAAACCGTCCTCGG |
|  | Reverse: ACAGTCGTGTGAGGA |
| ANGPTL4 mRNA  3’ end | Forward: GAGGCTGGTGGTTTGGCA |
|  | Reverse: ATGGGCTGGATCAACATGGT |
| PDK1 mRNA | Forward: ACCAGGACAGCCAATACAAG |
|  | Reverse: CCTCGGTCACTCATCTTCAC |
| RPL13A mRNA | Forward: CTCAAGGTCGTGCGTCTG |
|  | Reverse: TGGCTTTCTCTTTCCTCTTCTC |
| 18S rRNA | Forward: CGGCGACGACCCATTCGAAC |
|  | Reverse: GAATCGAACCCTGATTCCCCGTC |

**Supplementary Figure 1. TRIM28 and DNA-PK interact with HIF-1α.** (**A, B**) SUM159 cells were exposed to 20% or 1% O_2_ for 16 h, and ChIP assays were performed using antibody (Ab) against TRIM28, KU70, KU86, DNA-PKcs or HIF-1α. Primers encompassing the hypoxia response element (HRE) in the *ANGPTL4* or *PDK1* gene (A) and *LOX, LDHA, CA9, VEGFA, PKM2, SLC2A3, PGK1* or *PGF* gene (B) were used for qPCR, and non-HIF target gene *RPL13A* was analyzed as a negative control (A). The qPCR data were normalized to the result obtained at 20% O_2_. Values were expressed as the mean ± SEM of three biologically independent samples. ^**^*p* < 0.01, ^***^*p* < 0.001, ^****^*p* < 0.0001; ns, not significant (*p* > 0.05) by two-tailed Student’s t-test. Exact *p* values from left to right: < 0.0001, 0.0013, 0.0003, 0.0017, < 0.0001 (ANGPTL4); 0.0006, 0.0003, 0.0035, < 0.0001, 0.0001 (PDK1); 0.0896, 0.2929, 0.4353, 0.1753, 0.1127 (RPL13A); < 0.0001, < 0.0001, < 0.0001, 0.0004 (LOX); 0.0004, < 0.0001, < 0.0001, 0.0046 (CA9); 0.0002, 0.0001, < 0.0001, 0.0001 (PKM2); < 0.0001, < 0.0001, 0.0005, 0.0016 (PGK1); 0.0008, < 0.0001, < 0.0001, 0.001 (LDHA); < 0.0001, 0.0002, < 0.0001, 0.0017 (VEGFA); < 0.0001, < 0.0001, < 0.0001, < 0.0001 (SLC2A3); 0.0002, < 0.0001, 0.0003, 0.0005 (PGF). (**C**) The structure of myc-epitope-tagged HIF-1α deletion mutants are shown. (**D**) MDA-MB-231 cells were transfected with vector encoding a myc-tagged HIF-1α deletion mutant without (-) or with (+) an expression vector encoding FLAG-epitope-tagged KU86. Immunoprecipitation (IP) was performed using FLAG Ab followed by immunoblot assays.

**Supplementary Figure 2. Recruitment of TRIM28 and DNA-PKcs to HREs is HIF-1α-dependent.** SUM159 subclones expressing NTC or HIF-1α shRNA were exposed to 20% or 1% O_2_ for 16 h. ChIP was performed using antibody against TRIM28 (left panels) or DNA-PKcs (right panels) and qPCR was performed using primers specific for the *ANGPTL4* (upper panels) or *PDK1* (lower panels) HRE. The qPCR data were normalized to the result obtained for NTC cells at 20% O_2_. Values were expressed as the mean ± SEM of three biologically independent samples. ^**^*p* < 0.01, ^****^*p* < 0.0001 vs. NTC at 20% O_2_; ^##^*p* < 0.01, ^###^*p* < 0.001, ^####^*p* < 0.0001 vs. NTC at 1% O_2_ by two-way ANOVA Tukey's multiple comparisons. Exact *p* values from left to right: < 0.0001, < 0.0001, < 0.0001, 0.0006 (ANGPTL4); < 0.0001, < 0.0001, 0.0012, 0.0076 (PDK1).

**Supplementary Figure 3. Knockdown (KD) of TRIM28 and DNA-PKcs impairs HIF target gene expression. (A, B)** MDA-MB-231 cells were stably transfected with vector encoding non-targeting control (shNTC) short hairpin RNA (shRNA) or either of two different shRNAs targeting TRIM28 (A) or DNA-PKcs (B) and immunoblot assays were performed. **(C, D)** NTC and either TRIM28-KD (C) or DNA-PKcs-KD (D) subclones of MDA-MB-231 were exposed to 20% or 1% O_2_ for 24 hours and RT-qPCR analysis of ANGPTL4, PDK1, and RPL13A mRNA was performed. For each mRNA, qPCR data were normalized to the result obtained for shNTC cells at 20% O_2_. Values were expressed as the mean ± SEM of three biologically independent samples. ^****^*p* < 0.0001 vs. NTC at 20% O_2_; ^####^*p* < 0.0001 vs. NTC at 1% O_2_; ns, not significant (*p* > 0.05) by two-way ANOVA Tukey's multiple comparisons. Exact *p* values from left to right: < 0.0001 (C, ANGPTL4 and PDK1), 0.9909, 0.928, 0.9998 (C, RPL13A); < 0.0001 (D, ANGPTL4 and PDK1), 0.9793, > 0.999, 0.9989 (D, RPL13A). **(E)** RNA-seq data from SUM159 subclones were analyzed. *Left*: Gene ontology analysis revealed that hypoxia-induced genes that are dependent on HIF, TRIM28 and DNA-PKcs are enriched for genes involved in angiogenesis and glycolysis. *Right*: Volcano plot of TRIM28 and DNA-PKcs target genes in SUM159 cells exposed to 20% or 1% O_2_ for 24 hours (n = 3 biologically independent samples. Data were analyzed by Genialis using DESeq2 program, two-sided Wald test with multiple test correction, FDR < 0.05). **(F)** Venn analysis shows the overlap among hypoxia-repressed genes that are TRIM28-, DNA-PKcs- or HIF-dependent in SUM159 cells as determined by RNA-seq.

**Supplementary Figure 4. TRIM28 or DNA-PK knockdown impairs HIF occupancy of HREs but not HIF protein stability.** **(A)** NTC, TRIM28-KD and DNA-PKcs-KD subclones of MDA-MB-231 were exposed to 20% or 1% O_2_ for 16 h and ChIP assays were performed using HIF-1α, HIF-1β or HIF-2α Ab. Primers encompassing the HRE in the *ANGPTL4* or *PDK1* gene were used for qPCR and non-HIF target gene *RPL13A* was analyzed as a negative control. For each primer pair, the qPCR data were normalized to the result obtained for shNTC cells at 20% O_2_. Values were expressed as the mean ± SEM of three biologically independent samples. ^****^*p* < 0.0001 vs. NTC at 20% O_2_; ^###^*p* < 0.001, ^####^*p* < 0.0001 vs. NTC at 1% O_2_; ns, not significant (*p* > 0.05) by two-way ANOVA Tukey's multiple comparisons. Exact *p* values from left to right: < 0.0001, < 0.0001, < 0.0001, < 0.0001, < 0.0001, 0.0004, < 0.0001, < 0.0001, 0.0003 (ANGPTL4); < 0.0001, < 0.0001, < 0.0001, < 0.0001, < 0.0001, 0.0001, < 0.0001, < 0.0001, 0.0002 (PDK1); 0.4036, 0.9032, 0.9905, > 0.9999, > 0.9999, 0.5558, > 0.9999, 0.9998, 0.887 (RPL13A). **(B)** NTC, TRIM28-KD, KU70-KD, KU86-KD and DNA-PKcs-KD subclones of MDA-MB-231 were exposed to 20% or 1% O_2_ for 8 h and immunoblot assays were performed.

**Supplementary Figure 5. Analysis of KU70-KD and KU86-KD subclones of MDA-MB-231.** **(A)** MDA-MB-231 cells were stably transfected with vector encoding NTC shRNA (shNTC) or one of five different shRNAs targeting KU70 (shKU70 #1 to #5) and immunoblot assays were performed. **(B)** NTC and KU70-KD subclones were exposed to 20% or 1% O_2_ for 24 h and RT-qPCR assays were performed. For each mRNA, the qPCR data were normalized to the result obtained for shNTC cells at 20% O_2_. Values were expressed as the mean ± SEM of three biologically independent samples. ^****^*p* < 0.0001 vs. NTC at 20% O_2_; **^####^***p* < 0.0001 vs. NTC at 1% O_2_; ns, not significant (*p* > 0.05) by two-way ANOVA Tukey's multiple comparisons. Exact *p* values from left to right: < 0.0001 (ANGPTL4 and PDK1); > 0.999, 0.8225, 0.9207 (RPL13A). **(C)** MDA-MB-231 cells were stably transfected with vector encoding shNTC or one of five different shRNAs targeting KU86 (shKU86 #1 to #5) and immunoblot assays were performed. **(D)** NTC and KU86-KD subclones were exposed to 20% or 1% O_2_ for 24 h and RT-qPCR assays were performed. The qPCR data were normalized to the result obtained for shNTC cells at 20% O_2_. Values were expressed as the mean ± SEM of three biologically independent samples. ^****^*p* < 0.0001 vs. NTC at 20% O_2_; **^####^***p* < 0.0001 vs. NTC at 1% O_2_; ns, not significant (*p* > 0.05) by two-way ANOVA Tukey's multiple comparisons. Exact *p* values from left to right: < 0.0001 (ANGPTL4 and PDK1); > 0.999, 0.9981, 0.981 (RPL13A). **(E)** MDA-MB-231 cells were stably transfected with lentiviral vectors encoding shNTC or shRNA targeting TRIM28. The cells were exposed to 20% or 1% O_2_ for 8 h and IP was performed with KU86 Ab, followed by immunoblot assays.

**Supplementary Figure 6. Analysis of DNA damage response pathway. (A)** SUM159 cells were exposed to 1% O_2_ for 0, 15, 30, or 60 min and immunoblot assays were performed. **(B)** SUM159 cells were exposed to 20% or 1% O_2_ for 16 h, and ChIP assays were performed using the indicated antibody (Ab). Primers encompassing HRE sites in the *ANGPTL4* or *PDK1* gene were used for qPCR. For each primer pair, the qPCR data were normalized to the mean result at 20% O_2_. Values were expressed as the mean ± SEM of three biologically independent samples. ^**^*p* < 0.01, ^****^*p* < 0.0001; ns, not significant (*p* > 0.05) by two-tailed Student’s t-test. Exact *p* values from left to right: 0.4511, < 0.0001 (ANGPTL4); 0.6142, 0.001 (PDK1). **(C)** MDA-MB-231 cells were exposed to 20% or 1% O_2_ for 24 h in the presence of DMSO or KU55933 and RT-qPCR assays were performed. For each primer pair, the qPCR data were normalized to the mean result for DMSO-treated cells at 20% O_2_. Values were expressed as the mean ± SEM of three biologically independent samples. ^****^*p* < 0.0001 vs. NTC at 20% O_2_; ns, not significant (*p* > 0.05) by two-way ANOVA Tukey's multiple comparisons. Exact *p* values from left to right: < 0.0001, 0.8244, 0.999, < 0.0001, 0.7454, 0.9106.

**Supplementary Figure 7. TRIM28 phosphorylation at serine-824 (pS824) is required but E3 SUMO ligase activity is not required for HIF target gene expression.** **(A)** MDA-MB-231 cells were exposed to 20% or 1% O_2_ for 16 h and ChIP assays were performed using TRIM28 or pS824-TRIM28 Ab. Primers encompassing the *ANGPTL4* or *PDK1* gene HRE were used for qPCR and non-HIF target gene *RPL13A* was analyzed as a negative control. Results were normalized to NTC at 20% O_2_. Values were expressed as the mean ± SEM of three biologically independent samples. ^***^*p* < 0.001, ^****^*p* < 0.0001; ns, not significant (*p* > 0.05) by two-tailed Student’s t-test. Exact *p* values from left to right: 0.0001, < 0.0001 (ANGPTL4); < 0.0001, 0.0003 (PDK1); 0.4116, 0.5016 (RPL13A). **(B)** MDA-MB-231 TRIM28-KD subclones stably transfected with vector encoding TRIM28-WT or TRIM28-S824D were exposed to 20% or 1% O_2_ for 24 hours in the presence of vehicle (DMSO) or NU7441 (4 μM), followed by RT-qPCR analysis of ANGPTL4, PDK1 and RPL13A mRNA. For each mRNA, the qPCR data were normalized to the result obtained for DMSO-treated cells at 20% O_2_. Values were expressed as the mean ± SEM of three biologically independent samples. ^****^*p* < 0.0001 vs. TRIM28-WT (DMSO) at 20% O_2_; ^####^*p* < 0.0001 vs. TRIM28-WT (DMSO) at 1% O_2_; ^&&&&^*p* < 0.0001 vs. TRIM28-WT (NU7441) at 1% O_2_; ns, not significant (*p* > 0.05) by two-way ANOVA Tukey's multiple comparisons. Exact *p* values from left to right: < 0.0001, < 0.0001, < 0.0001, 0.0639 (ANGPTL4); < 0.0001, < 0.0001, < 0.0001, 0.2496 (PDK1); 0.0669, 0.079, 0.9217, 0.999 (RPL13A). **(C)** MDA-MB-231 shNTC subclone and shTRIM28 subclone stably transfected with empty vector (EV) or vector encoding TRIM28-WT (WT) or TRIM28-C65/68A (CA) were analyzed by immunoblot assay. **(D)** MDA-MB-231 subclones were exposed to 20% or 1% O_2_ and RT-qPCR assays were performed. For each mRNA, the qPCR data were normalized to the result obtained for NTC cells at 20% O_2_. Values were expressed as the mean ± SEM of three biologically independent samples. ^****^*p* < 0.0001 vs. NTC at 20% O2; ^####^*p* < 0.0001 vs. NTC at 1% O2; ^&&&&^*p* < 0.0001 vs. shTRIM28-EV at 1% O2 by two-way ANOVA Tukey's multiple comparisons. Exact *p* values: < 0.0001 (ANGPTL4 and PDK1).

**Supplementary Figure 8. TRIM28 and DNA-PKcs are required for release of paused Pol II and transcriptional elongation of HIF target genes in MDA-MB-231 cells.** **(A)** MDA-MB-231 cells stably transfected with lentiviral vector encoding NTC or shRNA targeting DNA-PKcs or TRIM28 were exposed to 20% or 1% O_2_ for 16 h, and ChIP was performed using Ab against Pol II-S5P, Pol II-S2P or total Pol II. Primers encompassing the *ANGPTL4* or *PDK1* HRE were used for qPCR and *RPL13A* was analyzed as a negative control. The qPCR data were normalized to the result obtained for NTC cells at 20% O_2_. Values were expressed as the mean ± SEM of three biologically independent samples. ^***^*p* < 0.001, ^****^*p* < 0.0001 vs. NTC at 20% O_2_; ^####^*p* < 0.0001 vs. NTC at 1% O_2_; ns, not significant (*p* > 0.05) by two-way ANOVA Tukey's multiple comparisons. Exact *p* values from left to right: < 0.0001, < 0.0001, < 0.0001, 0.839, < 0.0001, 0.4253, 0.9998, < 0.0001, 0.2472 (ANGPTL4); 0.0003, < 0.0001, < 0.0001, 0.9933, < 0.0001, 0.6841, 0.8802, < 0.0001, > 0.9999 (PDK1); 0.9383, 0.9109, 0.8951, 0.1165, 0.887, 0.1133, 0.0711, 0.7792, 0.076 (RPL13A). **(B)** MDA-MB-231 subclones expressing shNTC or shRNA targeting DNA-PKcs or TRIM28 were exposed to 20% or 1% O_2_ for 16 h and ChIP-qPCR assays using NELF-E Ab were performed. The qPCR data were normalized to the result obtained for NTC cells at 20% O_2_. Values were expressed as the mean ± SEM of three biologically independent samples. ^***^*p* < 0.001, ^****^*p* < 0.0001 vs. NTC at 20% O_2_; ^####^*p* < 0.0001 vs. NTC at 1% O_2_; ns, not significant (*p* > 0.05) by two-way ANOVA Tukey's multiple comparisons. Exact *p* values from left to right: < 0.0001, < 0.0001, < 0.0001 (ANGPTL4); 0.0003, < 0.0001, < 0.0001 (PDK1); 0.9872, 0.7747, 0.9952 (RPL13A). **(C)** MDA-MB-231 subclones were exposed to 20% or 1% O_2_ for 24 h and RT-qPCR assays were performed using primers that target the 5’ end, middle or 3’ end of ANGPTL4 mRNA. For each primer pair, the qPCR data were normalized to the result obtained for NTC cells at 20% O_2_. Values were expressed as the mean ± SEM of three biologically independent samples. ^**^*p* < 0.01, ^****^*p* < 0.0001 vs. NTC at 20% O_2_; ^####^*p* < 0.0001 vs. NTC at 1% O_2_; ns, not significant (*p* > 0.05) by two-way ANOVA Tukey's multiple comparisons. Exact *p* values from left to right: 0.0032, 0.0734, 0.0594 (5’ end), < 0.0001 (middle and 3’ end). (**D**) MDA-MB-231 subclones were exposed to 20% or 1% O_2_ for 16 hours and ChIP-qPCR assays were performed using Ab against H3K4me3, H3K36me3 or total H3. The qPCR data were normalized to the result obtained for NTC cells at 20% O_2_. Values were expressed as the mean ± SEM of three biologically independent samples. ^****^*p* < 0.0001 vs. NTC at 20% O_2_; ^####^*p* < 0.001 vs. NTC at 1% O_2_; ns, not significant (*p* > 0.05) by two-way ANOVA Tukey's multiple comparisons. Exact *p* values from left to right: < 0.0001, < 0.0001, 0.9254, > 0.9999, < 0.0001, 0.9997, 0.2097, < 0.0001, 0.8702 (PDK1); 0.999, 0.9109, 0.999, > 0.9999, 0.1552, 0.9974, 0.8427, 0.8743, 0.4035 (RPL13A).
